# Supplementary material for: Protein Scaffold‐Based Multimerization of Soluble ACE2 Efficiently Blocks SARS‐CoV‐2 Infection In Vitro and In Vivo
Source: Adv Sci (Weinh). 2022 Jul 27;9(27):2201294. doi: 10.1002/advs.202201294 (PMC9353362; doi:10.1002/advs.202201294)
Supplement: Supplementary file 1 — Supporting Information [file ADVS-9-2201294-s001.pdf]

ADVANCED SCIENCE
Open Access

Supporting Information

for *Adv. Sci.*, DOI 10.1002/advs.202201294

Protein Scaffold-Based Multimerization of Soluble ACE2 Efficiently Blocks SARS-CoV-2 Infection In Vitro and In Vivo

Alisan Kayabolen, Ugur Akcan, Doğancan Özturan, Hivda Ulbegi-Polat, Gizem Nur Sahin, Nareg Pinarbasi-Degirmenci, Canan Bayraktar, Gizem Soyler, Ehsan Sarayloo, Elif Nurtop, Berna Ozer, Gulen Guney-Esken, Tayfun Barlas, Ismail Selim Yildirim, Ozlem Dogan, Sercin Karahuseyinoglu, Nathan A. Lack, Mehmet Kaya, Cem Albayrak, Fusun Can, Ihsan Solaroglu and Tugba Bagci-Onder**

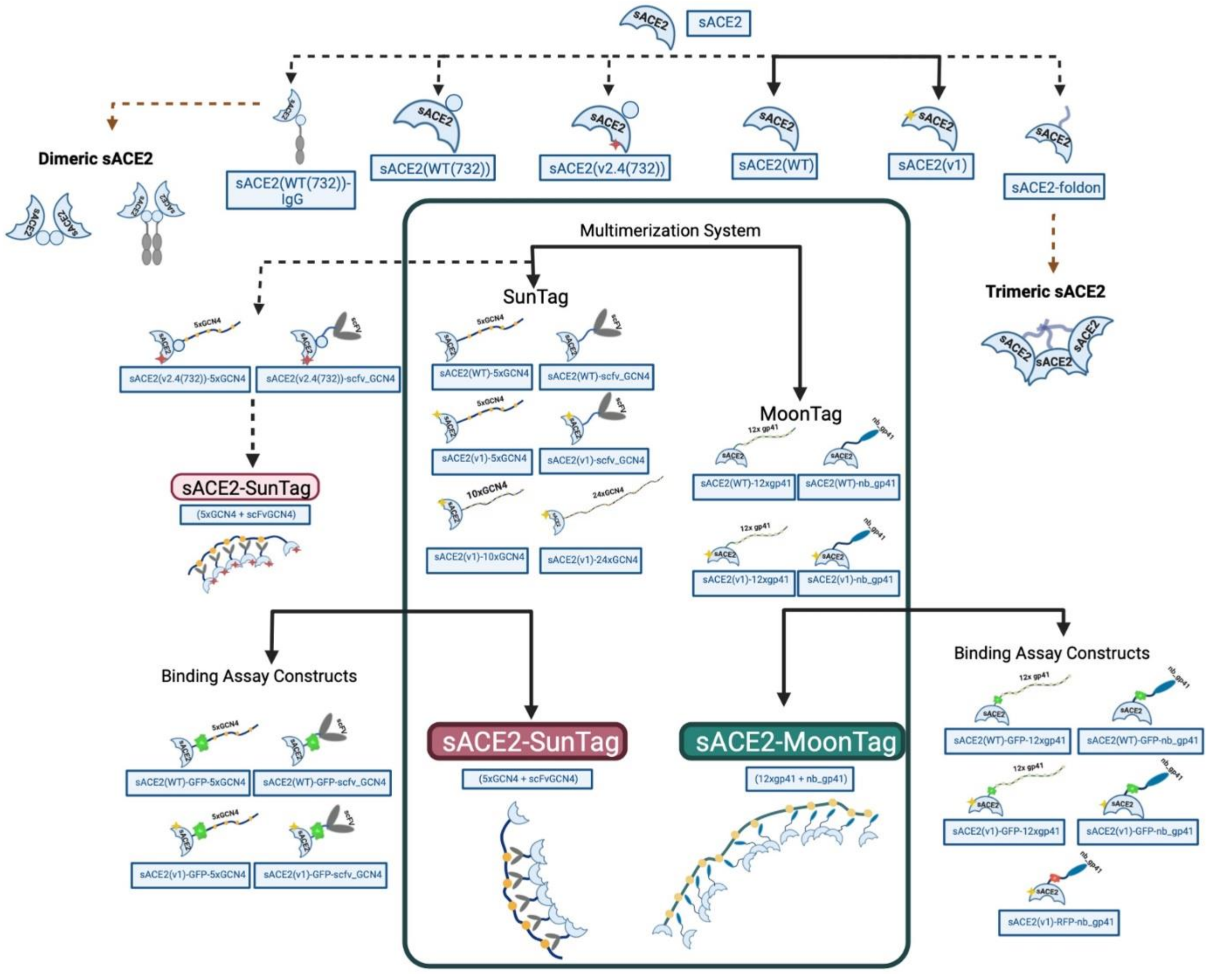

Figure S1. Representative models for each fusion protein generated/used in this study. *(Created with Biorender.com)*

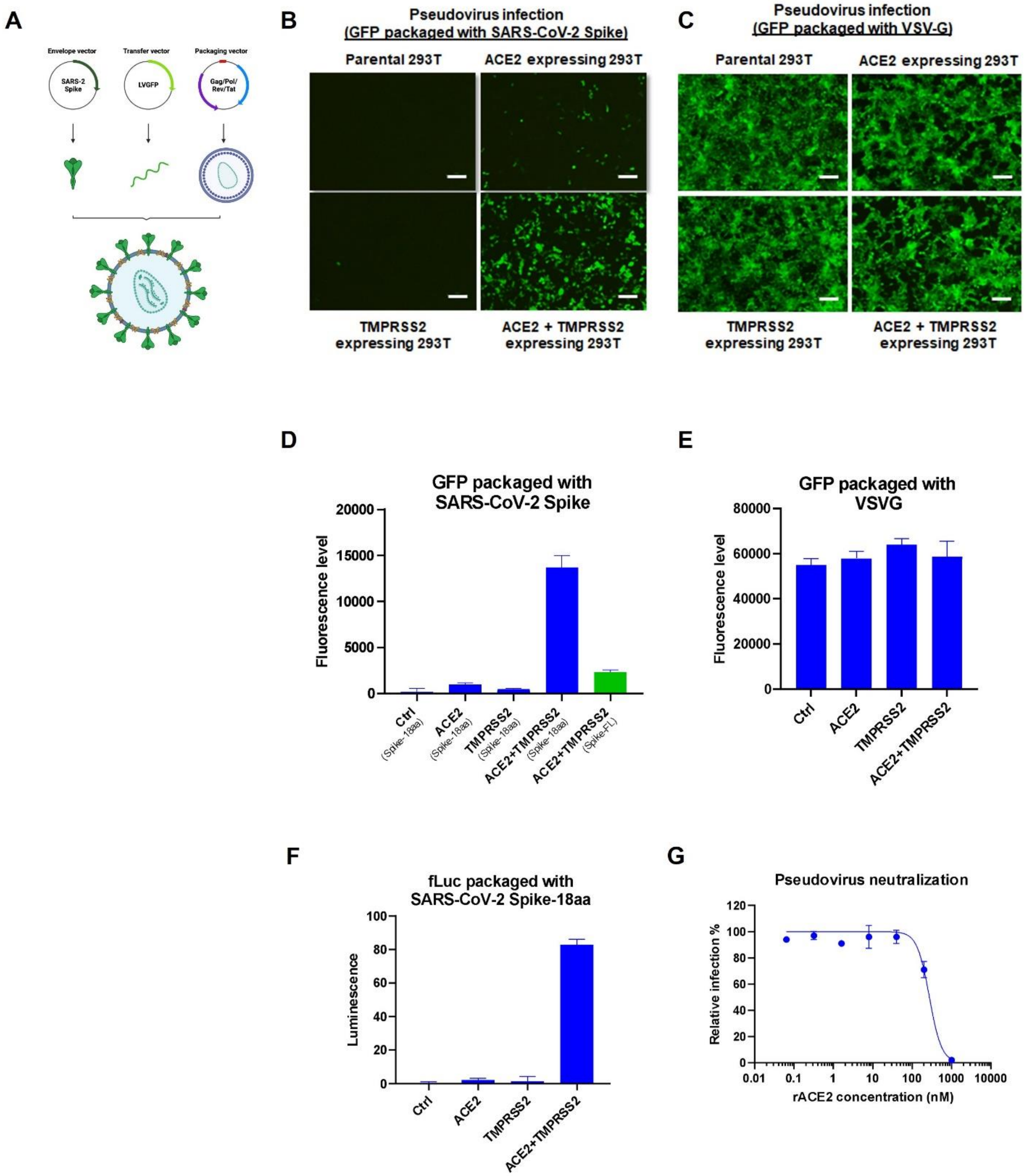

Figure S2. Optimization of pseudovirus infection and ACE2-based neutralization. A) Schematic representation of production of GFP-packaged pseudoviruses bearing SARS-CoV-2 Spike. *(Created with Biorender.com)* **B-C)** Microscopic images of infection of HEK293T cells expressing either ACE2 or TMPRSS2 individually, or ACE2+TMPRSS2 together, with GFP-packaged pseudoviruses bearing either SARS-CoV-2 Spike **(B)** or VSV-G **(C)** glycoproteins.

Scale bars = 100 μm. **D-E)** Quantification of infection with GFP-packaged pseudoviruses bearing SARS-CoV-2 Spike (18-aa deleted or full length) **(D)** or VSV-G **(E)** Parental HEK293T infection was used as control, and infection rates were normalized to fluorescence level of control. **F)** Quantification of infection with fLuc-packaged pseudoviruses bearing SARS-CoV-2 Spike. Parental HEK293T infection was used as control, and infection rates were normalized to luminescence level of control. **G)** Relative infection rate of ACE2 and TMPRSS2 expressing HEK293T cells with pseudoviruses bearing SARS-CoV-2 Spike (18-aa) in the presence of human recombinant ACE2 (rACE2).

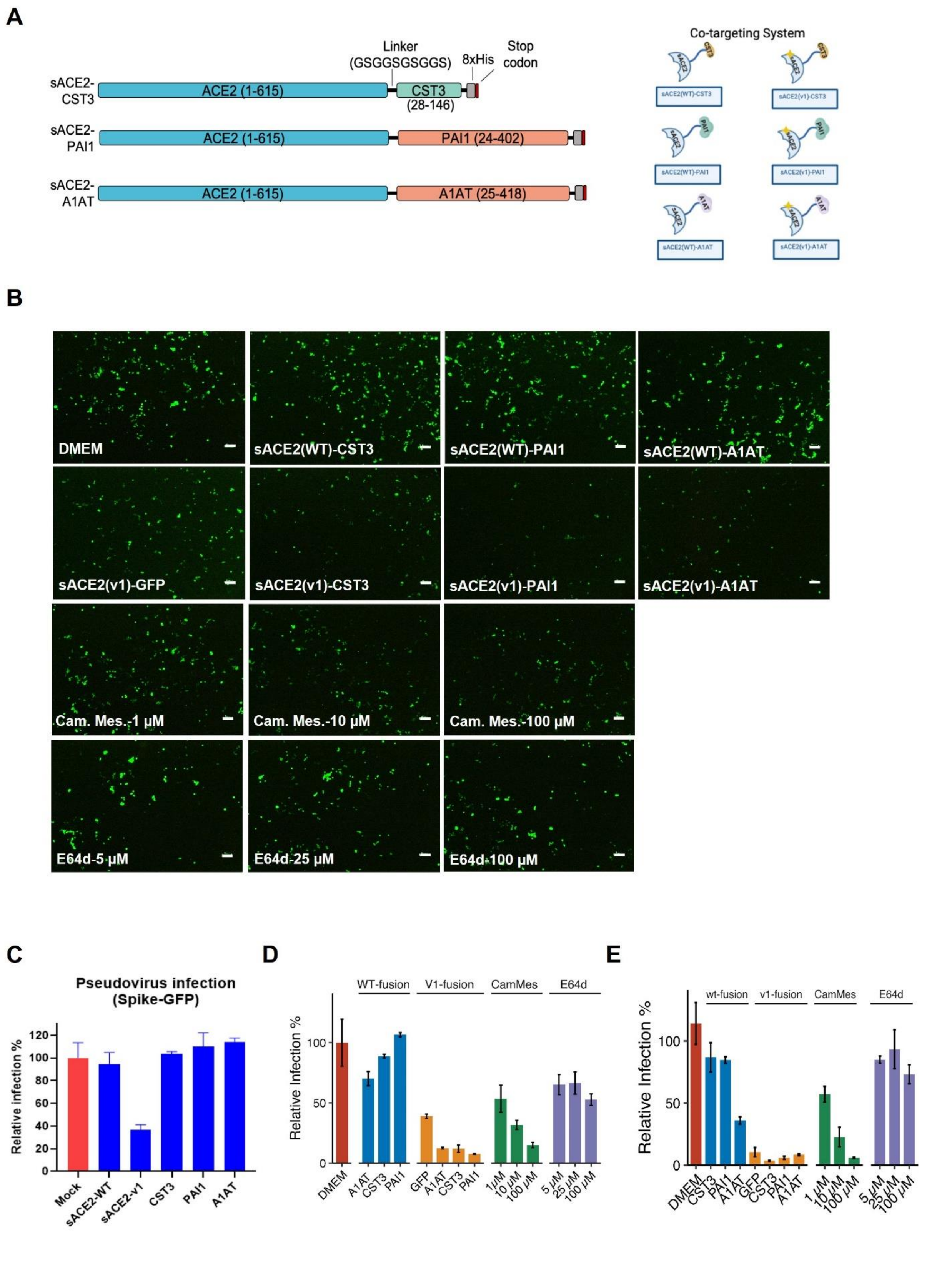

Figure S3. Neutralization of SARS-CoV-2 Spike-bearing pseudoviruses by sACE2-protease

inhibitor fusions. A) Schematic representations of sACE2-fusion constructs. **B)** Microscopic images of HEK293T cells infected with GFP-packaged pseudoviruses in the presence of conditioned media (CM) collected from sACE2-expressing or control cells, and chemicals camostat mesylate (CamMes) and E64d. Scale bar = 100 μm. **C)** Relative infection of HEK293T cells with GFP-packaged pseudoviruses in the presence of CM collected from cells expressing sACE2, or several proteases (CST3, PAI1, A1AT) individually. CM from mock transfected cells were used as control, and infection rates were calculated as relative fluorescence to control wells. **D-E)** Relative infection rate of HEK293T cells with GFP-packaged (**D**) or fLuc-packaged **(E)** pseudoviruses in the presence of CM collected from sACE2-protease inhibitor fusion expressing or mock transfected cells, and chemicals CamMes or E64d. CM from mock transfected cells were used as control, and infection rates were calculated as relative fluorescence to control wells.

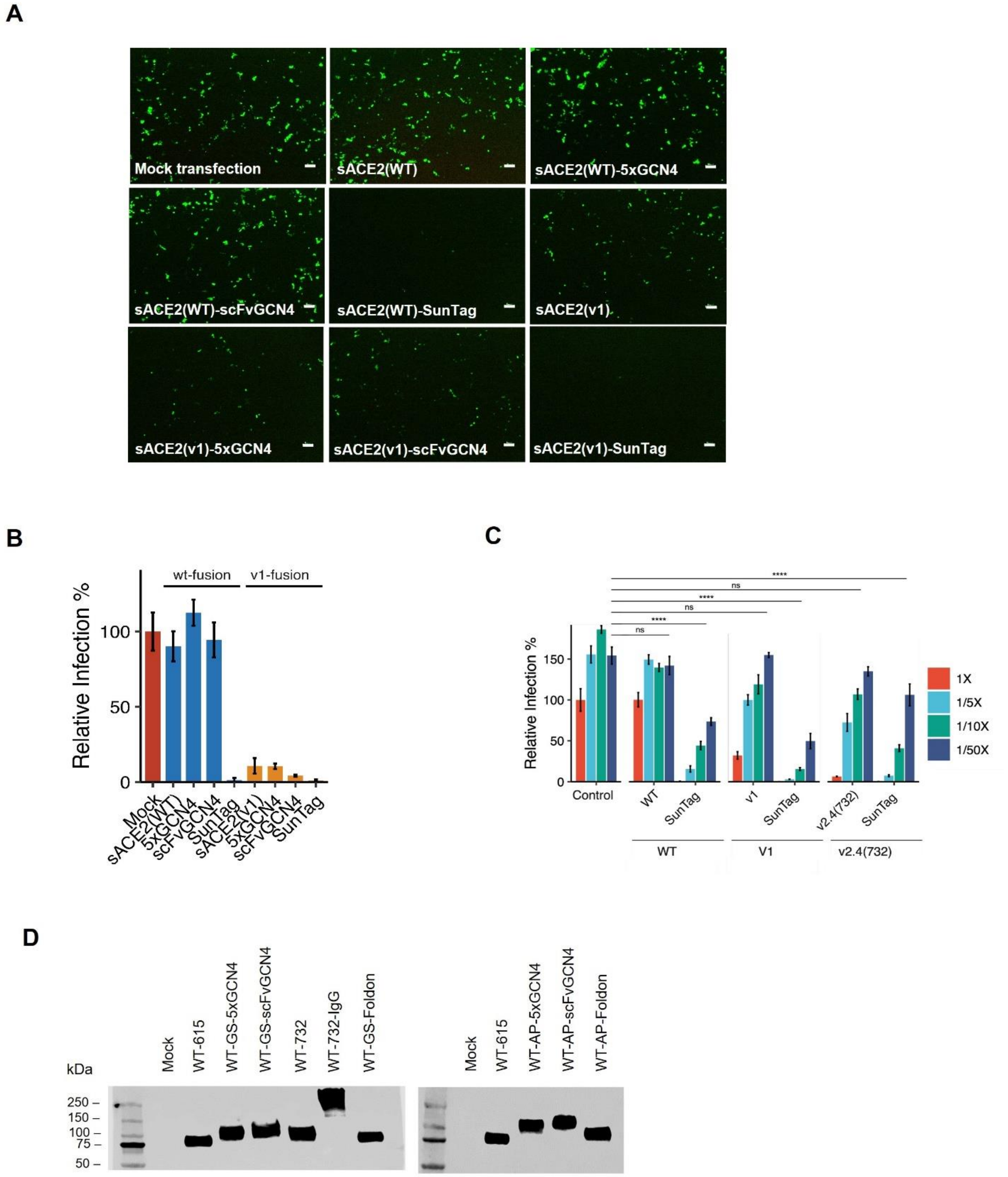

Figure S4. Neutralization of SARS-CoV-2 Spike-bearing pseudoviruses by multimeric sACE2. A) Microscopic images of HEK293T cells infected with GFP-packaged pseudoviruses in the presence of conditioned media (CM) collected from sACE2-expressing or control cells. Scale bar = 100 μm. **B)** Relative infection rate of HEK293T cells with fLuc-packaged

pseudoviruses in the presence of CM collected from sACE2-expressing or mock transfected cells. CM from mock transfected cells were used as control, and infection rates were calculated as relative to control wells. **C)** Relative infection with pseudovirus in the presence of different dilutions of CM containing different sACE2 constructs with or without SunTag. CM were diluted with DMEM up to 1/50X. CM from mock transfected cells were used as control, and infection rates were calculated as relative to control wells. **D)** Western blot images of CM collected from cells transfected with monomeric, dimeric, trimeric or multimeric sACE2 components having flexible (GS) or rigid (AP) linkers. Anti-ACE2 primary antibody was used for blotting.(ns: $p > 0.05$, *: $p <= 0.05$, **: $p <= 0.01$, ***: $p <= 0.001$, ****: $p <= 0.0001$, One way ANOVA.)

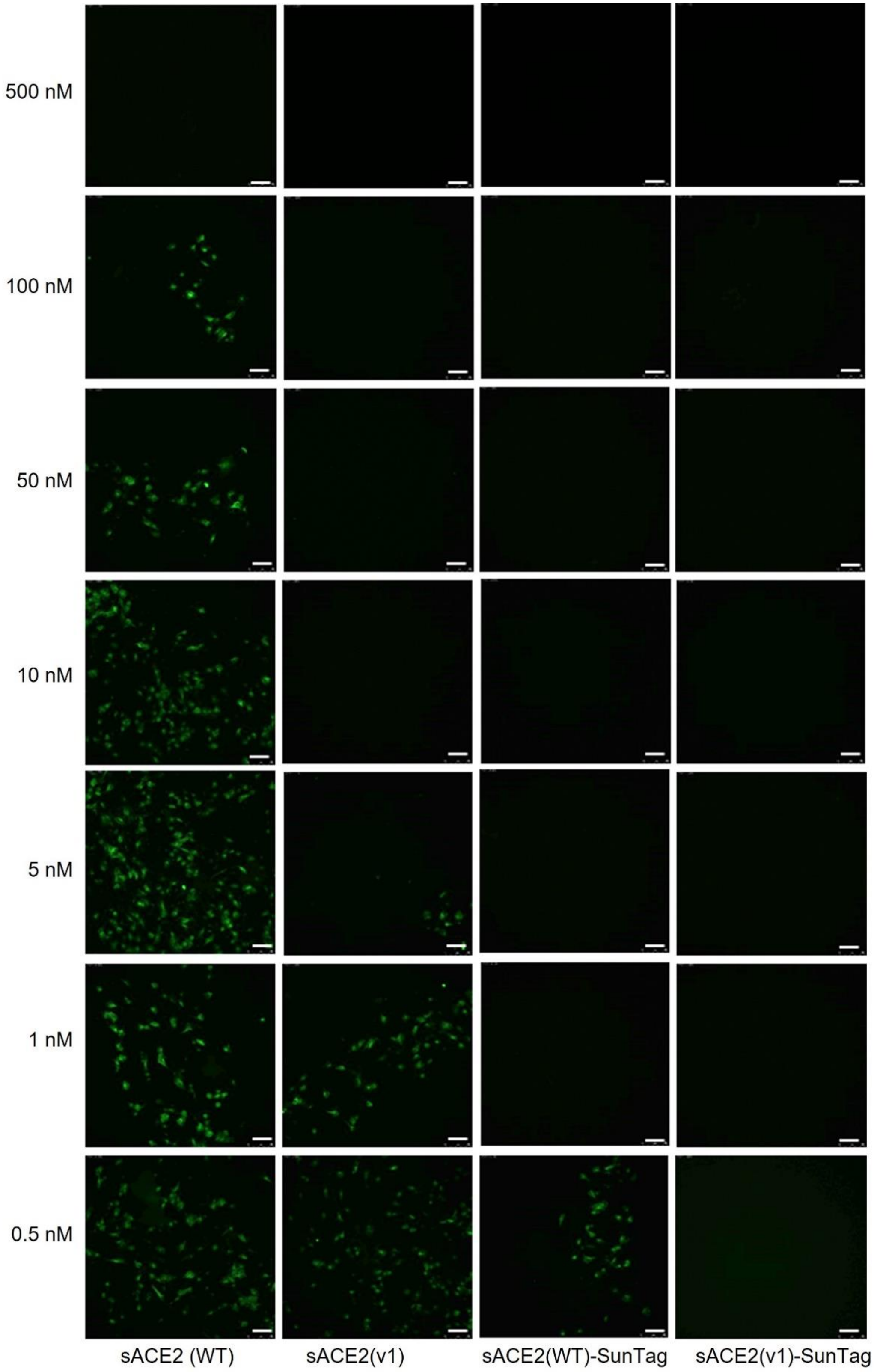

Figure S5. Neutralization assay with low dose SARS-CoV-2 isolate. Microscopic images of Vero-CCL81 cells infected with authentic SARS-CoV-2 (10^3 pfu/ml) upon incubation with

different concentrations of purified sACE2 proteins for 1h. Cells were immunostained with anti-SARS-CoV-2 spike primary antibody (Green) after 24h of infection. Scale bar = 100 μm.

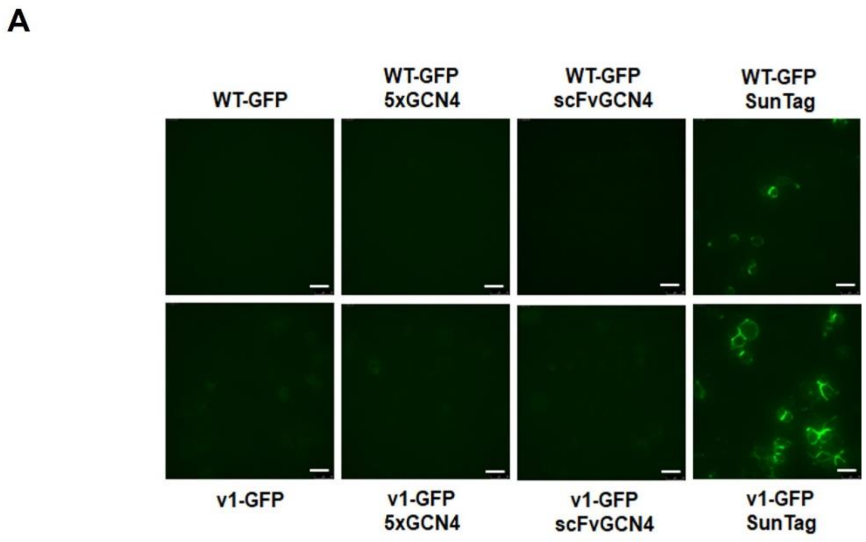

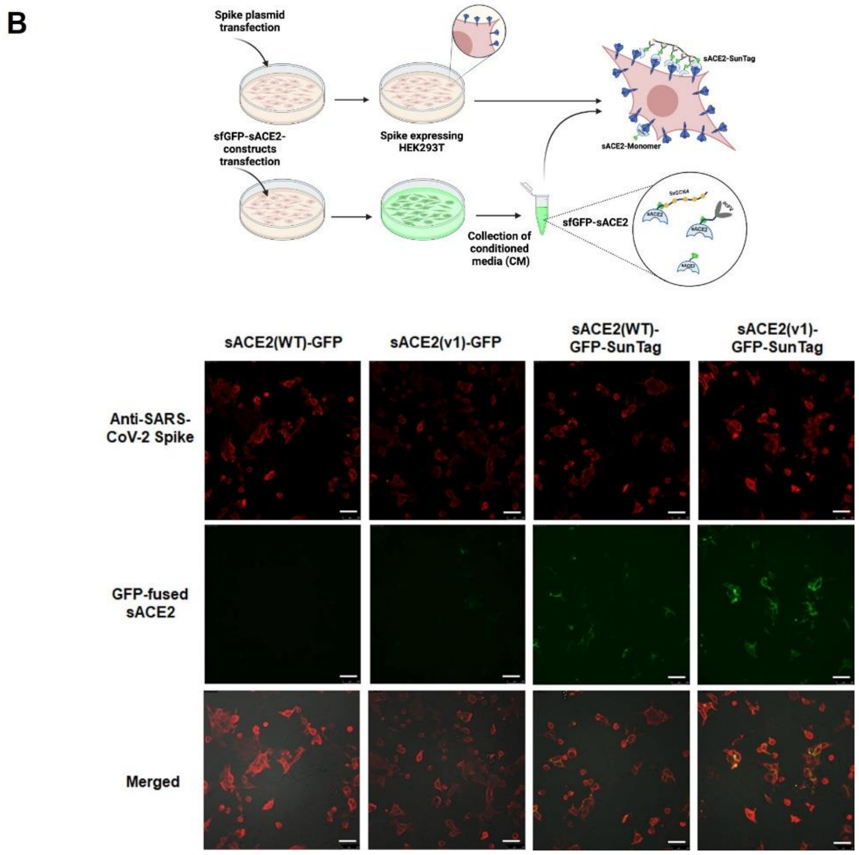

Figure S6. Spike-binding assay with GFP-fused sACE2 fusions. A) Microscopic images of Spike-expressing HEK293T cells upon incubation with CM that were collected from cells

transfected with sfGFP-fused sACE2(WT) or sACE2(v1) with individual or combined SunTag components. Scale bars = 50 μm. **B)** Microscopic images of HEK293T cells expressing SARS-CoV-2 Spike upon incubation with CM collected from sfGFP fused sACE2(WT) or sACE2(v1) with or without SunTag system. Cells were immunostained with anti-SARS-CoV-2 antibody (red). Scale bars = 50 μm. *(Created with Biorender.com)*

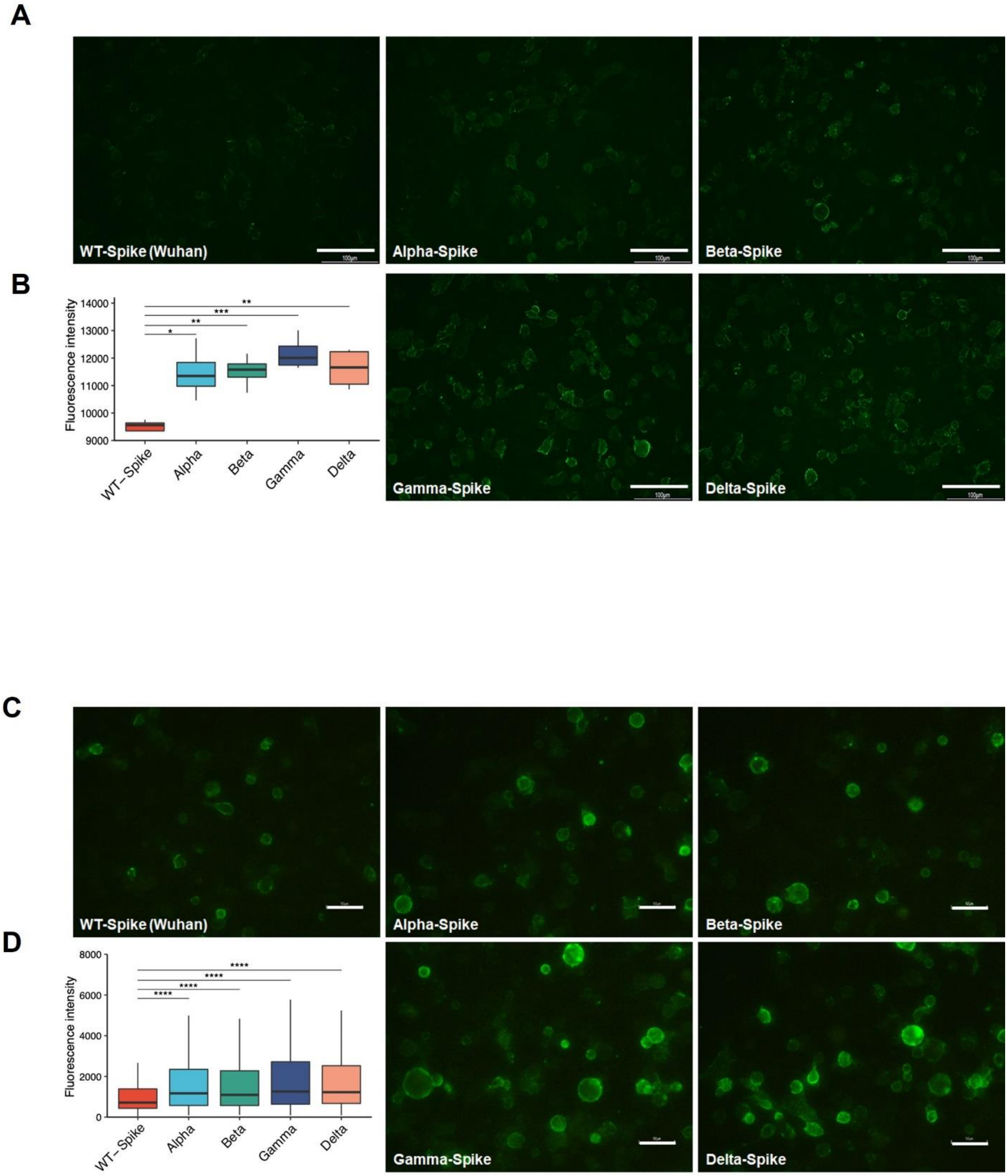

Figure S7. Variant-Spike binding assay with sACE2-MoonTag. Microscopic images of wild type or VOC Spike-expressing HEK293T cells upon incubation with conditioned media (CM) that were collected from cells transfected with sfGFP-fused version of sACE2(WT)-MoonTag. Scale bars = 100 μm. **B)** Quantification of Spike binding assay in (A). 4 images from each

condition were analyzed via ImageJ software. **C)** Microscopic images of wild type or VOC Spike-expressing HEK293T cells upon incubation with CM collected from cells transfected with sfGFP-fused version of sACE2(v1)-MoonTag. Scale bars = 50 μm. **D)** Quantification of Spike binding assay in (C). 4 images from each condition were analyzed via ImageJ software. (ns: $p > 0.05$, *: $p <= 0.05$, **: $p <= 0.01$, ***: $p <= 0.001$, ****: $p <= 0.0001$, Student's t-test.)

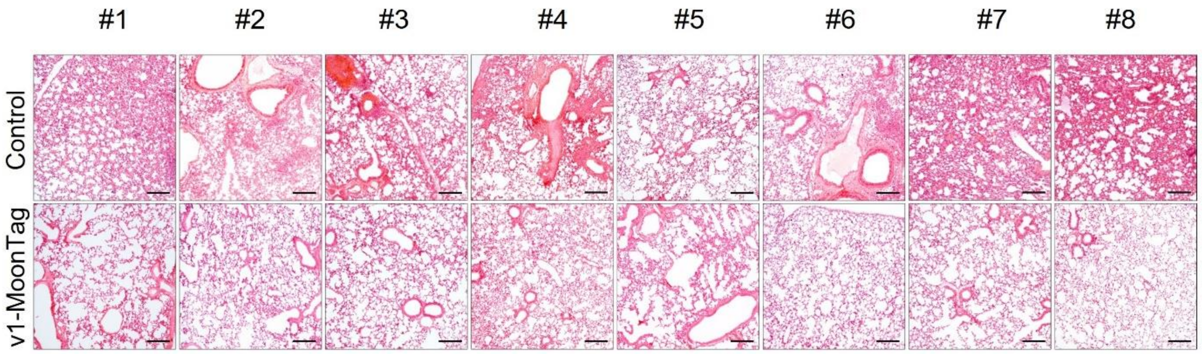

Figure S8. Representative histological images of lung sections harvested from each animal in the control or v1-MoonTag groups. Tissues obtained from v1-MoonTag groups have shown remarkable healing and restoration of tissue architecture in most of the animals. Scale bars=90 μm.

Vector ID	Insert	Insert Size (bp) (Ins1-Linker-Ins2)	Linker	Purif. Tag (C-term)	Mutations
pcDNA3.1_sACE2(WT)	sACE2(WT)	1845			
pcDNA3.1_sACE2(WT)-CST3	sACE2(WT)-CST3	1845+30+438	GSGGSGSGGS		
pcDNA3.1_sACE2(WT)-CST3-8h	sACE2(WT)-CST3	1845+30+438	GSGGSGSGGS	8xHis	
pcDNA3.1_sACE2(WT)-PAI1	sACE2(WT)-PAI1	1845+30+1137	GSGGSGSGGS		
pcDNA3.1_sACE2(WT)-PAI1-6h	sACE2(WT)-PAI1	1845+30+1137	GSGGSGSGGS	6xHis	
pcDNA3.1_sACE2(WT)-A1AT	sACE2(WT)-A1AT	1845+30+1182	GSGGSGSGGS		
pcDNA3.1_sACE2(WT)-A1AT-6h	sACE2(WT)-A1AT	1845+30+1182	GSGGSGSGGS	6xHis	
pcDNA3.1_sACE2(WT)-5xGCN4	sACE2(WT)-5xGCN4	1845+30+642	GSNGPTDAAE		
pcDNA3.1_sACE2(WT)-5xGCN4-8h	sACE2(WT)-5xGCN4	1845+30+642	GSNGPTDAAE	8xHis	
pcDNA3.1_sACE2(WT)-AP-5xGCN4-8h	sACE2(WT)-5xGCN4	1845+51+642	GSPAPAPAPAP APAPAP	8xHis	
pcDNA3.1_sACE2(WT)-scFvGCN4	sACE2(WT)-scFvGCN4	1845+30+741	GSGGSGSGGS		
pcDNA3.1_sACE2(WT)-scFvGCN4-8h	sACE2(WT)-scFvGCN4	1845+30+741	GSGGSGSGGS	8xHis	
pcDNA3.1_sACE2(WT)-AP-scFvGCN4	sACE2(WT)-5xGCN4	1845+51+741	GSPAPAPAPAP APAPAP	8xHis	
pcDNA3.1_sACE2(WT)-sfGFP-5xGCN4	sACE2(WT)-sfGFP-5xGCN4	1845+30+708+30+642	GSGGSGSGGS/ GSNGPTDAAE		
pcDNA3.1_sACE2(WT)-sfGFP-scFvGCN4	sACE2(WT)-sfGFP-scFvGCN4	1845+30+708+30+741	GSGGSGSGGS/ GSGGSGSGGS		
pcDNA3.1_sACE2(WT)-12xgp41-8h	sACE2(WT)-12xgp41	1845+30+705	GSGGSGSGSG	8xHis	
pcDNA3.1_sACE2(WT)-nb_gp41-8h	sACE2(WT)-nb_gp41	1845+30+366	GSGGSGSGGS	8xHis	
pcDNA3.1_sACE2(WT)-sfGFP-12xgp41-8h	sACE2(WT)-sfGFP-12xgp41	1845+30+708+30+705	GSGGSGSGGS/ GSGGSGSGSG	8xHis	
pcDNA3.1_sACE2(WT)-sfGFP-nb_gp41-8h	sACE2(WT)-sfGFP-nb_gp41	1845+30+708+30+366	GSGGSGSGGS/ GSGGSGSGGS	8xHis	
pcDNA3.1_sACE2(WT)-GS-foldon	sACE2(WT)-foldon	1845+30+81	GSGGSGSGGS		
pcDNA3.1_sACE2(WT)-AP-foldon	sACE2(WT)-foldon	1845+51+81	GSPAPAPAPAP APAPAP		
pcDNA3.1_sACE2(v1)	sACE2(v1)	1845			H34A, T92Q, Q325P, A386L
pcDNA3.1_sACE2(v1)-CST3	sACE2(v1)-CST3	1845+30+438	GSGGSGSGGS		H34A, T92Q, Q325P, A386L
pcDNA3.1_sACE2(v1)-CST3-6h	sACE2(v1)-CST3	1845+30+438	GSGGSGSGGS	6xHis	H34A, T92Q, Q325P, A386L
pcDNA3.1_sACE2(v1)-PAI1	sACE2(v1)-PAI1	1845+30+1137	GSGGSGSGGS		H34A, T92Q, Q325P, A386L
pcDNA3.1_sACE2(v1)-PAI1-6h	sACE2(v1)-PAI1	1845+30+1137	GSGGSGSGGS	6xHis	H34A, T92Q, Q325P, A386L
pcDNA3.1_sACE2(v1)-A1AT	sACE2(v1)-A1AT	1845+30+1182	GSGGSGSGGS		H34A, T92Q, Q325P, A386L
pcDNA3.1_sACE2(v1)-A1AT-6h	sACE2(v1)-A1AT	1845+30+1182	GSGGSGSGGS	6xHis	H34A, T92Q, Q325P, A386L
pcDNA3.1_sACE2(v1)-5xGCN4	sACE2(v1)-5xGCN4	1845+30+642	GSNGPTDAAE		H34A, T92Q, Q325P, A386L
pcDNA3.1_sACE2(v1)-5xGCN4-8h	sACE2(v1)-5xGCN4	1845+30+642	GSNGPTDAAE	8xHis	H34A, T92Q, Q325P, A386L
pcDNA3.1_sACE2(v1)-scFvGCN4	sACE2(v1)-scFvGCN4	1845+30+741	GSGGSGSGGS		H34A, T92Q, Q325P, A386L
pcDNA3.1_sACE2(v1)-scFvGCN4-8h	sACE2(v1)-scFvGCN4	1845+30+741	GSGGSGSGGS	8xHis	H34A, T92Q, Q325P, A386L
pcDNA3.1_sACE2(v1)-10xGCN4	sACE2(v1)-10xGCN4	1845+30+1398	GSGGSGSGGS		H34A, T92Q, Q325P, A386L
pcDNA3.1_sACE2(v1)-24xGCN4	sACE2(v1)-24xGCN4	1845+30+1782	GSGGSGSGGS		H34A, T92Q, Q325P, A386L
pcDNA3.1_sACE2(v1)-sfGFP-5xGCN4	sACE2(v1)-5xGCN4	1845+30+708+30+642	GSGGSGSGGS/ GSNGPTDAAE		H34A, T92Q, Q325P, A386L
pcDNA3.1_sACE2(v1)-sfGFP-scFvGCN4	sACE2(v1)-scFvGCN4	1845+30+708+30+741	GSGGSGSGGS/ GSGGSGSGGS		H34A, T92Q, Q325P, A386L
pcDNA3.1_sACE2(v1)-12xgp41-8h	sACE2(v1)-12xgp41	1845+30+705	GSGGSGSGSG	8xHis	H34A, T92Q, Q325P, A386L
pcDNA3.1_sACE2(v1)-nb_gp41-8h	sACE2(v1)-nb_gp41	1845+30+366	GSGGSGSGGS	8xHis	H34A, T92Q, Q325P, A386L
pcDNA3.1_sACE2(v1)-sfGFP-12xgp41-8h	sACE2(v1)-sfGFP-12xgp41	1845+30+708+30+705	GSGGSGSGGS/ GSGGSGSGSG	8xHis	H34A, T92Q, Q325P, A386L
pcDNA3.1_sACE2(v1)-sfGFP-nb_gp41-8h	sACE2(v1)-sfGFP-nb_gp41	1845+30+708+30+366	GSGGSGSGGS/ GSGGSGSGGS	8xHis	H34A, T92Q, Q325P, A386L
pcDNA3.1_sACE2(v1)-mRFP-nb_gp41-8h	sACE2(v1)-mRFP-nb_gp41	1845+30+672+30+366	GSGGSGSGGS/ GSGGSGSGGS	8xHis	H34A, T92Q, Q325P, A386L
pcDNA3.1_sACE2v2.4(732)-5xGCN4-8h	sACE2v2.4(732)-5xGCN4	2193+30+642	GSNGPTDAAE	8xHis	T27Y, L79T, N330Y
pcDNA3.1_sACE2v2.4(732)-scFvGCN4-8h	sACE2v2.4(732)-scFvGCN4	2193+30+741	GSGGSGSGGS	8xHis	T27Y, L79T, N330Y
SARS-CoV-2 Spike-Alpha (C-term. 18-aa trunc.)	SARS-CoV-2 Spike	3759			ΔH69V70, N501Y, D614G, P681H
SARS-CoV-2 Spike-Beta (C-term. 18-aa trunc.)	SARS-CoV-2 Spike	3765			K417N, E484K, N501Y, D614G
SARS-CoV-2 Spike-Gamma (C-term. 18-aa trunc.)	SARS-CoV-2 Spike	3765			K417T, E484K, N501Y, D614G
SARS-CoV-2 Spike-Delta (C-term. 18-aa trunc.)	SARS-CoV-2 Spike	3765			L452R, T478K, D614G, P681R
SARS-CoV-2 Spike-Omicron (C-term. 18-aa trunc.)	SARS-CoV-2 Spike	3756			A67V, ΔH69/V70, T95I, G142D, V143-, Y144-, Y145-, N211-, L212I, +214EPE, G339D, S371L, S373P, S375F, K417N, N440K, G446S, S477N, T478K, E484A, Q493R, G496S, Q498R, N501Y, Y505H, T547K, D614G, H655Y, N679K, P681H, N764K, D796Y, N856K, Q954H, N969K, L981F

Table S1. Details of vectors generated in this study
